# The Added-Value of Using Participatory Approaches to Assess the Acceptability of Surveillance Systems: The Case of Bovine Tuberculosis in Belgium

**DOI:** 10.1371/journal.pone.0159041

**Published:** 2016-07-27

**Authors:** Clémentine Calba, Flavie Luce Goutard, Luc Vanholme, Nicolas Antoine-Moussiaux, Pascal Hendrikx, Claude Saegerman

**Affiliations:** 1 CIRAD, UPR AGIRs, F-34398 Montpellier, France; 2 CIRAD, UPR AGIRs, 10900 Bangkok, Thailand; 3 Kasetsart University, 10900 Bangkok, Thaïlande; 4 DG Control Policy, Federal Agency for the Safety of the Food Chain (FASFC), Brussels, Belgium; 5 Tropical Veterinary Institute, Faculty of Veterinary Medicine; 6 French Agency for Food, Environmental and Occupational Health Safety (ANSES), Lyon, France; 7 Research Unit of Epidemiology and Risk Analysis applied to Veterinary Sciences (UREAR-ULg),Fundamental and Applied Research for Animal and Health (FARAH), Faculty of Veterinary Medicine, University of Liege (ULg), Liège, Belgium; University of Minnesota, UNITED STATES

## Abstract

**Context and Objective:**

Bovine tuberculosis (bTB) surveillance in Belgium is essential to maintain the officially free status and to preserve animal and public health. An evaluation of the system is thus needed to ascertain the surveillance provides a precise description of the current situation in the country. The evaluation should assess stakeholders’ perceptions and expectations about the system due to the fact that the acceptability has an influence on the levels of sensitivity and timeliness of the surveillance system. The objective of the study was to assess the acceptability of the bTB surveillance in Belgium, using participatory tools and the OASIS flash tool (‘*analysis tool for surveillance systems*’).

**Methods:**

For the participatory process, focus group discussions and individual interviews were implemented with representatives involved with the system, both from cattle and wildlife part of the surveillance. Three main tools were used: *(i)* relational diagrams associated with smileys, *(ii)* flow diagrams associated with proportional piling, and *(iii)* impact diagrams associated with proportional piling. A total of six criteria were assessed, among which five were scored on a scale from -1 to +1. For the OASIS flash tool, one full day meeting with representatives from stakeholders involved with the surveillance was organised. A total of 19 criteria linked to acceptability were scored on a scale from 0 to 3.

**Results and Conclusion:**

Both methods highlighted a medium acceptability of the bTB surveillance. The main elements having a negative influence were the consequences of official notification of a bTB suspect case in a farm, the low remuneration paid to private veterinarians for execution of intradermal tuberculin tests and the practical difficulties about the containment of the animals. Based on the two evaluation processes, relevant recommendations to improve the surveillance were made. Based on the comparison between the two evaluation processes, the added value of the participatory approach was highlighted.

## Introduction

Bovine tuberculosis (bTB) is one of the most important livestock diseases worldwide and eradication remains an important challenge with global perspectives despite all efforts already made and measures taken over the last decades [1, 2]. This zoonotic disease caused by *Mycobacterium bovis* represents a constant (re-)emerging threat both for animal and human health, and has consequences for intracommunity and international trade of animals [3]. Indeed, this bacterium can infect a wide range of animal species, either domestic or wild, making the eradication of the disease very challenging [2, 4–6]. Moreover, the infection in cattle mostly appears without any clinical sign, meaning that the disease might go unnoticed for several years [3, 5]. The infection in cattle is most commonly detected in apparently healthy animals by a cellular immunological response to bovine tuberculin injection [7].

Guaranties for bovine tuberculosis have to be provided for trade of bovine animals in the European Union (EU) since 1964 (EU Directive 64/432/EEC). Several EU members states and some regions became officially tuberculosis free (OTF), meaning that the annual herd prevalence is below 0.1% for several consecutive years [8]. Belgium obtained the OTF status in 2003 by Decision 2003/467/EC [9]. Despite this OTF status, some sporadic outbreaks still occurred over the last years: one in 2011, one in 2012 and nine in 2013. In 2014, no outbreak was detected [10]. The objectives of the cattle surveillance system are to early detect any new case of the disease and to confirm the OTF status.

In some member states, presence of wildlife has been identified as an important risk factor for transmission of bovine tuberculosis in cattle. Indeed, *M*. *bovis* can infect a wide range of wild animals, which may be maintenance or spill-over hosts, and which may contaminate cattle either by direct or indirect contact [5]. Until now, bTB infection has never been detected in wild animals since the start in 2002 of wildlife surveillance in Belgium [9, 11].

Surveillance of bTB, both in cattle and in wildlife, is essential to follow-up the animal health situation and to maintain the Belgian OTF status, but also to protect public health from this zoonotic disease. Due to the economic importance for Belgium to maintain the OTF status, there is a need to evaluate the quality of the evidence provided by the system by estimating its sensitivity. Surveillance systems designed to prove freedom of disease require a higher sensitivity than systems designed to assess the prevalence of an endemic disease. Sensitivity is thus the essential measure of surveillance systems efficacy in supporting a claim to disease freedom [12, 13]. Moreover, due to the fact that one of the objectives of bTB surveillance is the early detection of sporadic new cases, there is also a need to assess the timeliness of the system. The quality of these two attributes may be impacted by the quality of other evaluation attributes, especially by the acceptability of the surveillance by all stakeholders [14]. Therefore it is essential to assess stakeholders’ willingness to participate in the surveillance in order to limit under-reporting by not notifying suspected cases, but also to identify ways to improve the current surveillance [15]. In addition, the acceptability has been listed by the Centers for Disease Control and Prevention (CDC) of the United-States as one of the main requirements for efficient surveillance [16].

Currently, the assessment of acceptability remains challenging due to a lack of clarity related to which aspects of this attribute to take into consideration and how to evaluate them [17]. Therefore, we propose to assess this evaluation attribute using a range of participatory methods and tools on one hand, and the OASIS flash tool on the other hand (acronym for the French translation of ‘analysis tool for surveillance systems’) [18].

The participatory methods and tools were proposed for evaluation due to the fact that perceptions and expectations of stakeholders regarding surveillance are critical elements to be considered in order to evaluate the acceptability of a system [19, 20]. This approach, based on visualisation tools and open discussions with all stakeholders, allows participants to play an active role in the definition and in the analysis of problems encountered during the mandatory participation to a surveillance programme, but also to find solutions to these problems [14, 21–24]. The use of participatory methods and tools allows collecting information to be used to assess the acceptability of the system, but also to get information related to the general context in which surveillance is implemented [25]. Moreover, through an iterative process (i.e. providing feedback to respondents), it allows stakeholders to propose a range of recommendations to improve the system [25]. The OASIS flash tool was proposed because it has been recognised efficient to evaluate animal health surveillance systems, and because this is the only ready-to-use tool available for the evaluation of animal health surveillance systems [26]. This tool was indeed implemented to evaluate different surveillance systems in France (e.g. Amat *et al*., 2015 [27]). By comparing these two methods of assessing acceptability, the objective was to highlight the added value of using participatory approach in the evaluation framework.

## Material and Methods

This study belongs to semi-quantitative research and does not concern human health and medical research or animal research. Hence, no ethics committee was consulted for study approval. Nonetheless, the approval to implement this work was obtained from the Belgian Chief Veterinary Officer. Furthermore, all ethics and principles of responsible research were observed at all investigation stages. The principal investigator carried out all interviews after presenting the study objectives and obtaining verbal informed consent from all participants. The privacy rights of participants were fully protected and all data were anonymized.

### Description of the surveillance system under evaluation

Surveillance of bTB in Belgium targets both cattle and wildlife. The surveillance of these two populations is the competence of different authorities; thus the coordination of surveillance is implemented by different organisations for cattle and wildlife populations. These organisations share information on animal diseases, including bTB, during an annual meeting implemented by the FASFC.

#### Cattle surveillance

**Fig 1:**
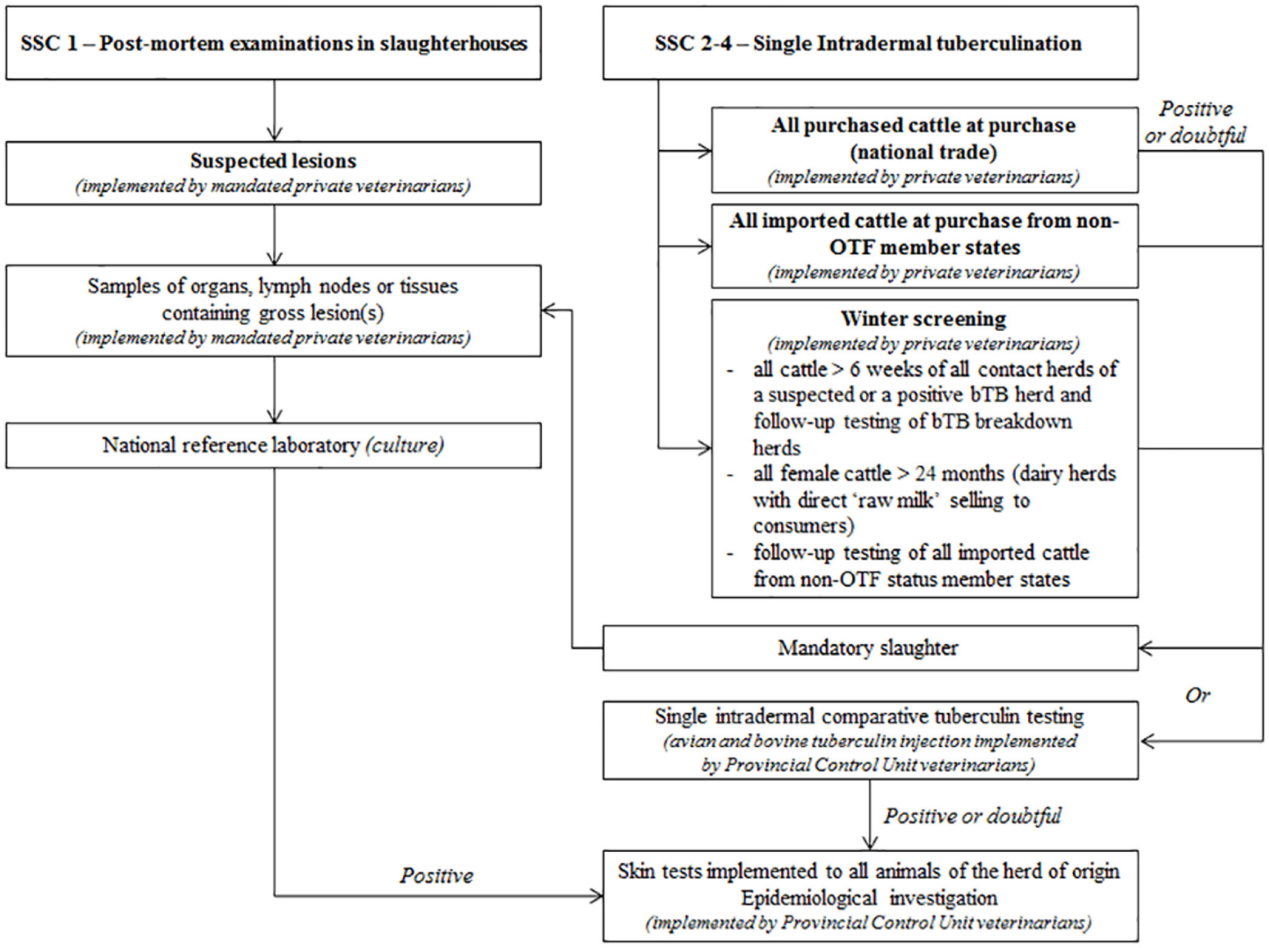
Description of the reporting system for cattle surveillance of bovine tuberculosis in Belgium.

The surveillance of cattle is implemented at national level and coordinated by the Belgian Federal Agency for the Safety of the Food Chain (FASFC). The system consists of four surveillance system components (SSCs) (Fig 1) [9]. The first SSC is implemented at slaughterhouse level, by systematic post-mortem examinations of all slaughtered bovines to detect gross bTB suspected lesions on organs and carcasses [3, 9]. The three other components are based on the use of SIT [28, 29]. SIT is implemented at individual animal level for any newly purchased animal by national, intracommunity or international trade (imports). Animals introduced within intracommunity trade from non-officially free member states or imports (from non-European countries) are supplementary tested by SIT during winter for three consecutive years. SIT is performed by private farm veterinarians who are mandated by the competent authority [28]. These private veterinarians receive financial rewards from the authority to implement the SIT.

Any positive or doubtful SIT result has to be reported to the Provincial Control Unit (PCU) of the FASFC. Official veterinarians of PCU will decide to re-test the animals by single intradermal comparative tuberculin testing (SICTT by avian and bovine tuberculin injection) or to mandatory slaughter the reactor animal for additional laboratory diagnosis. When suspected lesions are detected at post-mortem examination, samples of organs, lymph nodes or tissues containing gross lesion(s) are sent to the national reference laboratory for analysis. If a suspicion is confirmed by culture (i.e. *M*. *bovis* isolation), skin tests are implemented to all animals of the herd of origin and an epidemiological investigation is performed by PCU staff [30].

#### Wildlife surveillance

Wildlife surveillance is a competence of the Brussels, Walloon and Flemish regions. Due to the fact that wildlife populations are more concentrated in southern Belgium (Wallonia), the study was especially conducted in this region. The wildlife surveillance targets a range of diseases as well as bTB. In Wallonia, the surveillance is coordinated by the Faculty of Veterinary Medicine of the University of Liège and consists in two SSCs [11].

**Fig 2:**
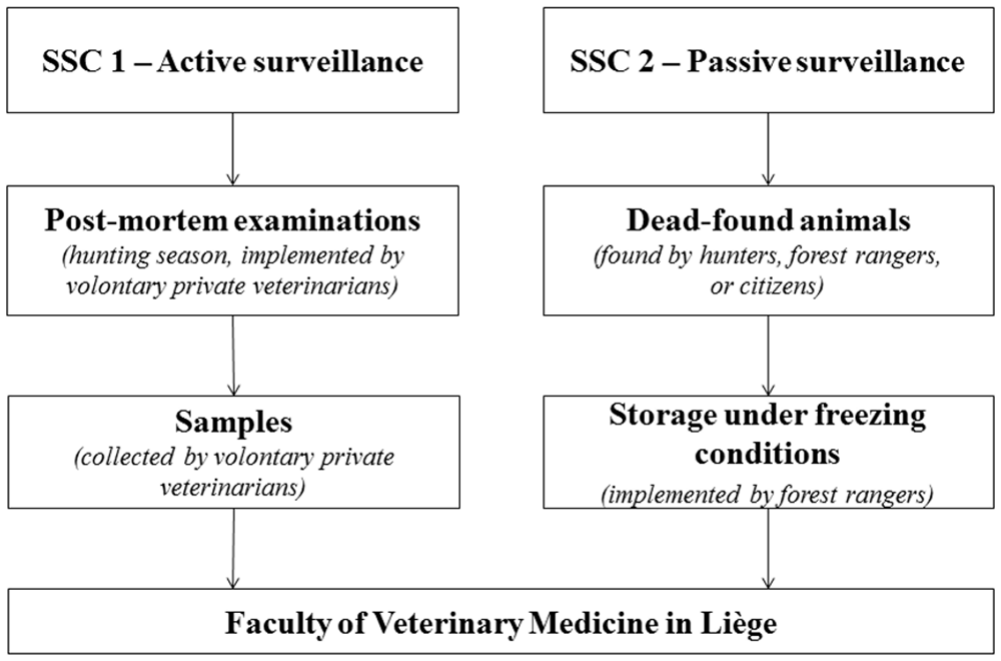
Description of the reporting system for wildlife surveillance of bovine tuberculosis in Belgium.

The active SSC targets cervids, wildboars and anatids. During hunting season some private veterinarians perform post-mortem examination at hunting parties on hunted wildlife species (Fig 2). These private veterinarians volunteer to perform these examinations and receive financial rewards to do so. After completion of a standard questionnaire, blood and tissues samples of some hunted wild animals are collected and sent to the Faculty of Veterinary Medicine in Liège for further analysis. The passive SSC targets a wide range of species, including ungulates, lagomorphs and carnivores. This surveillance is performed on dead-found animals, which can be collected all over the year by hunters, forest rangers, and even citizens. The cadavers are stored under freezing conditions (20 depots all over Wallonia) by forest rangers, and afterwards transmitted to the Faculty of Veterinary Medicine in Liège where a standardised procedure for necropsy examination is realised [11].

### Assessing acceptability using participatory approaches

#### Description of the method

Within the framework of the RISKSUR project (http://www.fp7-risksur.eu/), which aims to develop decision supporting tools for the design of cost-effective risk-based surveillance systems, a participatory method was developed to assess the acceptability of animal health surveillance systems [25]. Within this method, acceptability assessment is based on the following criteria: *(i)* the acceptability of the objective(s) of the system, *(ii)* the satisfaction of the role and the representation of the stakeholders’ utility in surveillance, *(iii)* the satisfaction of the consequences of the flow of information (i.e. changes in the activities and management at herd level following a suspicion or an outbreak), *(iv)* the satisfaction of the relations between different stakeholders, and *(v)* the trust in the system to fulfil its objectives. Another criterion was also used: the trust in the stakeholders involved in the bTB surveillance. Nevertheless, this criterion was not used to directly assess the acceptability of the system, but to provide explanatory information related to the trust attributed to the system.

To evaluate all those criteria the following procedure has been applied. *(i)* Identification of the stakeholders’ professional network and assessment of the satisfaction of the relations among them, through the elaboration of relational diagrams and the use of smileys. *(ii)* Representation of the information flow within the system and assessing the trust devoted to the system to fulfil its objectives, with the use of flow diagrams associated with proportional piling. *(iii)* Assessment of the satisfaction of the information flow (i.e. positive and negative impacts following a suspicion) with the use of impact diagrams associated with proportional piling. This methodological approach is presented in detail in Calba *et al*. (2015) [25].

#### Stakeholders involved in the evaluation

The objective was to include each type of stakeholders involved in both of the bTB surveillance systems. For the cattle surveillance, the aim was to involve *(i)* farmers (working with different types of farming: dairy, beef or mixed herds), *(ii)* private veterinarians (including those working at the slaughterhouses), *(iii)* experts of the national reference laboratory, *(iv)* representatives of the PCU, *(v)* representatives of the FASFC (headquarter), and *(vi)* representatives of the Federal Public Service (FPS) of public health, safety of the food chain and environment. For the wildlife surveillance system, the aim was to involve *(i)* hunters, *(ii)* forest rangers, and *(iii)* the surveillance system coordinator.

Focus group discussions and individual interviews were implemented between September 2014 and February 2015 by a single facilitator. All discussions during the interviews were recorded using an electronic device, in consent with the respondents.

#### Data analysis and outputs

Once the work in the field was completed, the discussions were subsequently transcribed in a Microsoft Word document (Microsoft Office 2010, Redmond, WA 98052–7329, USA), pictures of the diagrams were taken and data resulting in the implementation of smileys and proportional pilings were compiled in a Microsoft Excel file (Microsoft Office 2010, Redmond, WA 98052–7329, USA).

A thematic analysis was implemented on the data set using the R-based Qualitative Data Analysis package (RQDA). Themes were developed in a deductive way, based on the elements of the acceptability to be assessed. For each theme, specific codes were developed in an inductive way creating useful categories, based on a latent analysis. Reading and coding of the transcripts was repeated several times until no new codes were identified. This coding allowed the identification of useful categories used to convert the data set into semi-quantitative data following the scoring criteria developed from a previous study [25]. Additional scoring criteria were developed to assess the satisfaction of the relations among stakeholders as presented in Table 1.

**Table 1:** Semi-quantitative evaluation criteria used to assess the satisfaction of the relations between stakeholders involved in the surveillance system.

	Criteria			Final associated scores	
	*Satisfaction*	*Initial scores*	*Mean*		
**Relations between stakeholders**	Not at all satisfied	-2			
	Not satisfied	-1	[-2; -0,7]	Weak	-1
	Moderately satisfied	0	[-0,7; 0,7]	Medium	0
	Fairly satisfied	1	[0,7; 2]	Good	+1
	Very satisfied	2			

### OASIS flash evaluation process

#### Description of the method

OASIS flash is a standardized semi-quantitative assessment tool which was developed for the assessment of surveillance systems on zoonoses and animal diseases. This tool is based on a detailed questionnaire used to collect information to describe the operation of the system under evaluation. The information collected is synthetized through a list of criteria describing the situation and the operation of the surveillance system (78 criteria in total). These criteria are then scored on a scale from 0 to 3, following a scoring guide [18]. In the original OASIS, an evaluation team is responsible of the whole process which is implemented by visiting and interviewing a panel of local and national stakeholders of the surveillance, completing the detailed questionnaire, gathering a panel of stakeholders responsible for scoring the evaluation criteria and writing an evaluation report. The flash version of OASIS, which was used in this study, is skipping the interview of local and national stakeholders. The completion of the questionnaire is then performed by national experts who have a good knowledge of the surveillance system and the scoring of the evaluation criteria is performed by a selected panel of stakeholders.

The questionnaire was completed based on the available documentation. The scoring grid was pre-scored by external evaluators (3 persons). The grid was then presented to a panel of experts during a full day meeting, which should be representative of most of the stakeholders involved in the bTB surveillance. The objective of the meeting was to assign to each criterion a global score by consensus of all experts and to agree on comments (score justification, gap identification) among gathered experts.

#### Data analysis and outputs

Within the OASIS tool, once the scoring process is completed, the scores are combined and weighted to produce three graphical outputs. *(i)* A table showing the 10 different sections of the surveillance system (objectives and scope; central institutional organisation; field institutional organisation; diagnostic laboratory; surveillance tools; surveillance procedures; data management; training; restitution and diffusion of information; evaluation and performance) with a pie chart representing the corresponding compiled scores for each section. *(ii)* A histogram showing the scoring of seven critical control points that were developed by Dufour (1999) [31]. And finally *(iii)* a radar chart displaying the score of 10 of the evaluation attributes recommended by CDC and WHO [32]: *(i)* simplicity, *(ii)* flexibility, *(iii)* data quality, *(iv)* acceptability, *(v)* sensitivity, *(vi)* positive predictive value, *(vii)* representativeness, *(viii)* timeliness, *(ix)* stability and *(x)* usefulness [17]. To assess the acceptability, 19 criteria were taken into account with various weights applied to each one according to the strength of their links to acceptability of surveillance.

### Comparison between the two evaluation processes

The two approaches used to assess the acceptability of the bTB surveillance system in Belgium were based on a semi-quantitative process. With participatory approaches 6 evaluation criteria were considered, among which 5 were scored on a scale from -1 to +1. With the OASIS flash tool 19 criteria were considered, scored on a scale from 0 to 3. Some criteria were similar between these two approaches (n = 7). Some others were slightly different, but similar information could be collected (n = 5). Finally, some criteria were specific to each approach: 7 were specific to the OASIS flash tool, 2 to the process by participatory approach. These similarities and differences are presented in the Table 2.

**Table 2:** Comparison of the criteria used to assess acceptability with participatory approaches and with the OASIS flash tool.

	OASIS criteria	Participatory approaches criteria / Stakeholders
**Similar indicators**	- Taking partners’ expectations related to the objective into account	- Acceptability of the objective / All
	- Effective integration of laboratories in the surveillance system	- Acceptability of the operation of the surveillance system—Satisfaction of its own role / National reference laboratory
	- Simplicity of the notification procedure	- Acceptability of the operation of the surveillance system—Satisfaction of its own role / Private veterinarians—Hunters—Forest rangers
	- Simplicity of the data collection procedure	- Acceptability of the operation of the surveillance system—Satisfaction of its own role / Private veterinarians—Hunters—Forest rangers
	- Acceptability of the consequences of a suspicion or case for the source or collector of data	- Acceptability of the operation of the surveillance system—Satisfaction with the consequences of the information flow / Farmers—Private veterinarians—Hunters—Forest rangers
	- Feedback of the individual analyses results to field actors	- Acceptability of the operation of the surveillance system—Satisfaction with the relations / Farmers—Private veterinarians—Hunters—Forest rangers
	- Systematic feedback of the surveillance results to field actors (excluding news bulletin)	- Acceptability of the operation of the surveillance system—Satisfaction with the relations / Farmers—Private veterinarians—Hunters—Forest rangers
**Slightly different indicators**	- Frequency of meetings of the central coordinating body	- Acceptability of the operation of the surveillance system—Satisfaction with the relations / PCU—National reference laboratory—FASFC—FPS
	- Active role of intermediary units in the functioning of the system (validation, management, feedback)	- Acceptability of the operation of the surveillance system—Satisfaction of its own role / PCU—Forest rangers
		- Acceptability of the operation of the surveillance system—Satisfaction of the relations / Farmers—Private veterinarians—FASFC—Hunters—Wildlife coordinator
	- Adequacy of material and financial resources of intermediary units	- Acceptability of the operation of the surveillance system—Satisfaction of its own role / PCU—Forest rangers
	- Existence of coordination meetings at the intermediate level	- Acceptability of the operation of the surveillance system—Satisfaction of the relations / Farmers—Private veterinarians—Hunters
	- Adequacy of material and financial resources at the field level	- Acceptability of the operation of the surveillance system—Satisfaction of its own role / Private veterinarians—Hunters—Forest rangers
**Specific indicators**	- Existence of an operational management structure (central unit)	- Trust given to the system / All
	- Existence of an operational steering structure that is representative of the partners (steering committee)	- Trust given to other stakeholders involved in surveillance / All
	- Organization and operations of the system laid down in regulations, a charter, or a convention established between the partners	
	- Simplicity of the case or threat definition	
	- Adequacy of the data management system for the needs of the system (relational database, etc.)	
	- Initial training implemented for all field agents when joining the system	
	- Regular reports and scientific papers publications on the results of the surveillance	

PCU: Provincial Control Unit; FASFC: Federal Agency for the Safety of the Food Chain (headquarter); FPS: Federal Public Service health, food safety and environment

The results were compared regarding *(i)* the level of acceptability obtained by each approach and *(ii)* the main factors having an influence on this level.

## Results

### Participatory approaches process

#### Stakeholders involved

For the cattle surveillance system, 22 stakeholders were interviewed using 4 focus group discussions and 4 individual interviews. Among these stakeholders, 8 were farmers, 7 were private veterinarians, 2 were representatives from the national reference laboratory, one was a representative from the PCU, 2 were representatives from the FASFC and 2 from the FPS (Table 3).

**Table 3:** Stakeholders interviewed for the assessment of the acceptability of the bovine tuberculosis surveillance systems (i.e. cattle surveillance, wildlife surveillance) in Belgium.

	Stakeholders	Number	Type of interview (number)
**Cattle surveillance**	Farmers	8	Focus group discussions (3)
	Private veterinarians	7	Focus group discussion (1) Individual interviews (3)
	National reference laboratory	2	Focus group discussion (1)
	PCU	1	Individual interview (1)
	FASFC & FPS	2 + 2	Focus group discussion (1)
**Wildlife surveillance**	Hunters	7	Individual interviews (7)
	Forest rangers	4	Focus group discussion (1) Individual interview (1)
	System coordinator	1	Individual interview (1)
	**Total**	**34**	**20**

PCU: Provincial Control Unit; FASFC: Federal Agency for the Safety of the Food Chain (headquarter); FPS: Federal Public Service health, food safety and environment

For the wildlife surveillance, 12 stakeholders were interviewed using one focus group discussions and 9 individual interviews: 7 hunters were involved, 4 forest rangers and the system coordinator (Table 3).

#### Acceptability assessment

**Fig 3:**
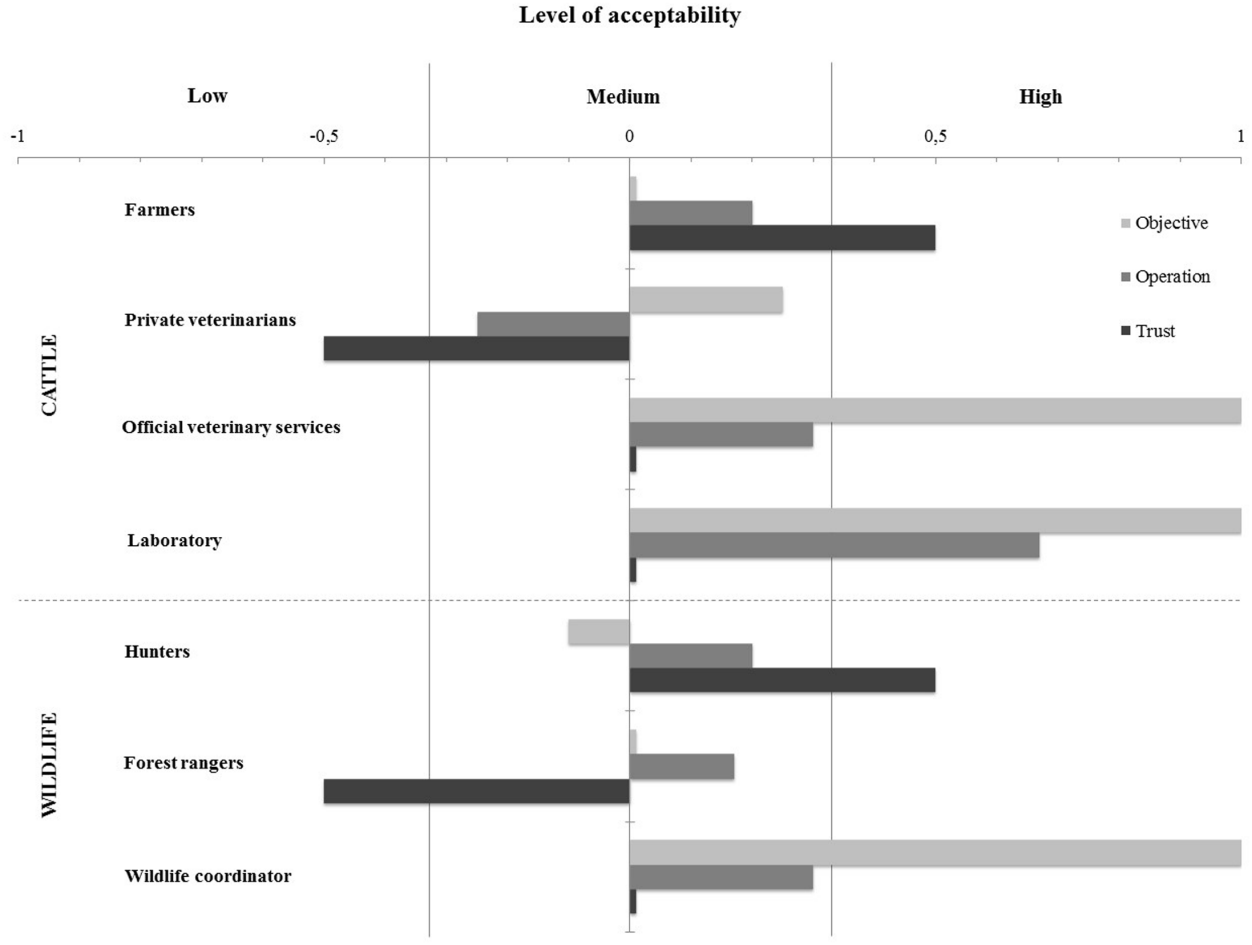
Graphical representation of each stakeholder groups’ mean level of acceptability of the bovine tuberculosis surveillance system in Belgium.

**Fig 4:**
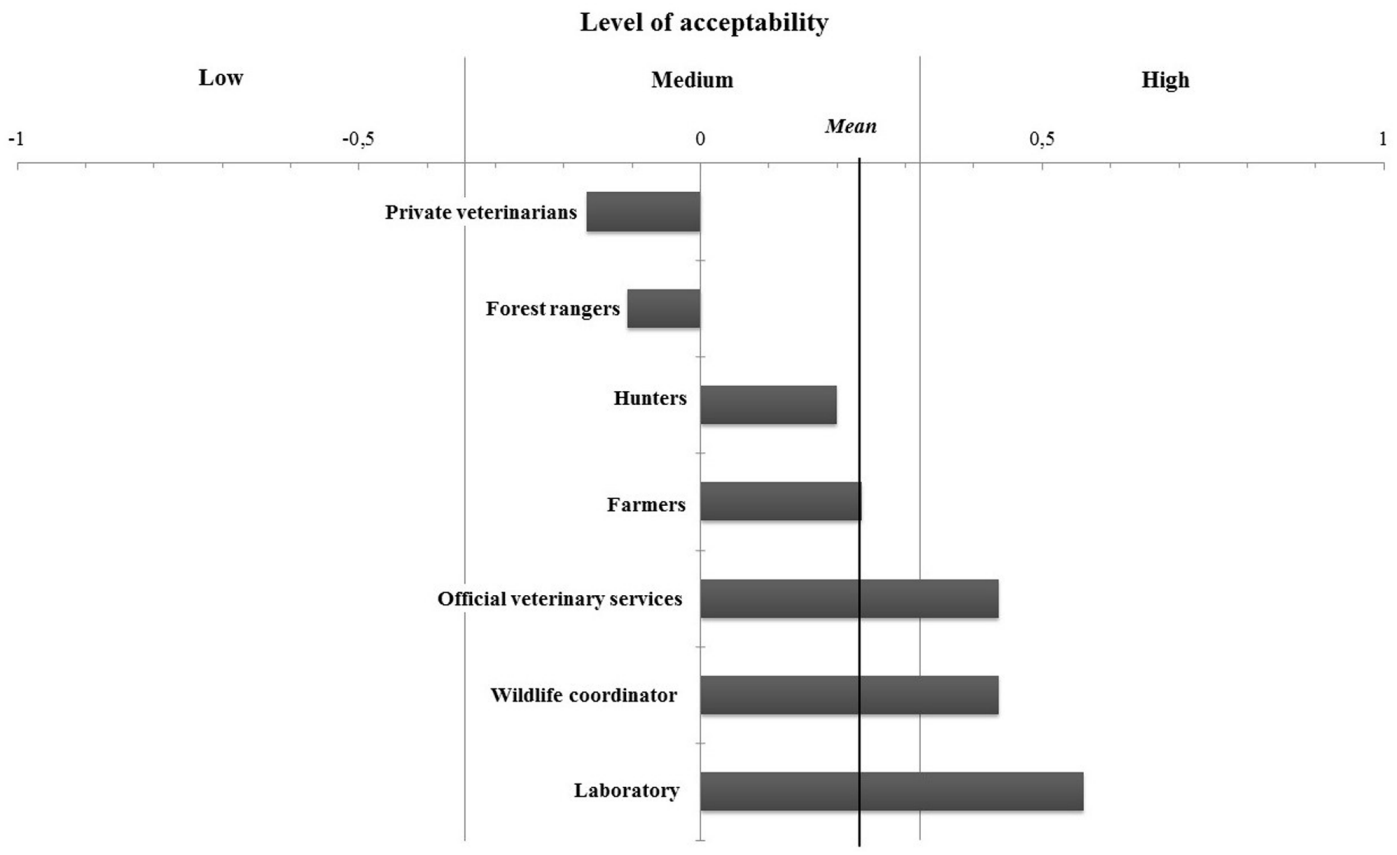
Graphical representation of the results obtained for the assessment of the acceptability of cattle and wildlife bovine tuberculosis surveillance systems in Belgium for each element (objective, operation and trust).

Each criterion was scored using the data collected during the interviews. Results showed a medium acceptability of the systems with a general mean of 0.23 (min/max = -0.33/+0.67). Results for each group of stakeholders are presented in Fig 3 regarding the mean level of acceptability, and in Fig 4 regarding the level of acceptability for each element.

Four groups of stakeholders had a medium acceptability of the system. The lowest acceptability was for private veterinarians and forest rangers, with respective means of -0.17 and -0.11; and then for hunters and farmers, with respective means of 0.2 and 0.24. The other stakeholders had a good acceptability of the system: the official veterinary services (0.44), the wildlife surveillance coordinator (0.44) and the national reference laboratory experts (0.56) (Fig 3).

#### Acceptability of cattle surveillance

The acceptability of the objective of the surveillance system (i.e. the primary reason for a surveillance system [33]) was medium for farmers (0) and private veterinarians (0.25), whereas it was good for representatives of the authorities (i.e. PCU, FASFC, FPS) (1) and for experts of the national reference laboratory (1) (Fig 4). The main objective of the surveillance for farmers and for private veterinarians was to safeguard animal health. None of the farmers, and only one group of private veterinarians (4 participants) knew about the OTF status. In contrast, this objective was clearly known and agreed by the laboratory staff and the official veterinary services.

The acceptability of the operation of the surveillance system (i.e. the surveillance process) was medium for farmers (0.2), for private veterinarians (-0.25) and for official veterinary services (0.3); whereas it was good for representatives of the national reference laboratory (0.67) (Fig 4).

Farmers were satisfied about their role in the surveillance but not with the consequences of the information flow. They stated that a suspicion would increase their workload and would generate mistrust between neighbouring farmers. They were satisfied about their relations with other stakeholders involved in surveillance, even if they highlighted some major issues with the official veterinary services (FASFC). Indeed, all of the groups stated that their controls are too strict: *‘In many cases*, *official inspectors of the FASFC have to find an infringement by their controls and to report that*. *To be not bothered*, *we have to make voluntary mistakes*. *That is pretty serious’* (focus group with farmers, 10^th^ November 2014).

Private veterinarians were not satisfied with their role in the system. They highlighted important constraints related to the implementation of the SIT, due to the fact that most of the farmers do not have good containment systems. The main problem for all private veterinarians was that they are caught between their clients and the official veterinary services: *‘When we observe doubtful reactions after a SIT*, *we always are under pressure of the client not to declare these results*, *because the farmer will be in stuck*. *[…] We are both judging and judged’* (individual interview with a private veterinarian, 1^st^ December 2014). This was impacting their satisfaction with the information flow due to communication problems with farmers and to the risk of losing their client. Nonetheless, one group of veterinarians highlighted, at some point, they would be satisfied to notify a doubtful or positive reactor to prove that their job is done *‘properly’* (focus group with private veterinarians, 6^th^ November 2014). Private veterinarians were satisfied with their relations with other stakeholders involved in the surveillance, even if they highlighted issues related to the relations with the official veterinary services. They found it regrettable that the official services do not get them more detailed information. They also deplored the lack of communication following a declaration of a suspicion, due to the fact that official services were going directly to their clients’ farm without informing them: *‘We do not get the information at the same moment as others despite we are the surveillance main actors’* (focus group with private veterinarians, 6^th^ November).

Representatives from the national reference laboratory were satisfied about their role in the surveillance system and did not identify any positive or negative consequences, at their level, following a suspicion. They were not completely satisfied about their relations with other stakeholders, especially with the FAFSC mainly due to the complexity of the structure of this Agency.

Official veterinarians were satisfied with their role in the surveillance, and did not identify any positive or negative consequences following a suspicion, due to the fact that dealing with a suspicion is *‘routine’* (focus group with representatives from the FASFC and FPS, 12^th^ November 2014). Official veterinarians were not completely satisfied about their relations with other stakeholders. They stated that it was complicated to take into consideration every actors’ expectations, and that some private veterinarians could complain when losing a client because of notifying unfavourable results of SIT.

The trust in the surveillance system (i.e. the confidence in the reliability of the system) was weak for the private veterinarians (-0.5); it was medium for the authorities (0) and for experts of the national reference laboratory (0); and good for farmers (0.5) (Fig 4). In summary, most of the respondents highlighted problems with the implementation of the SIT, interpretation of SIT results and highlighted the fact that private veterinarians are under pressure of their client.

#### Acceptability of wildlife surveillance

The acceptability of the objective of the surveillance system was medium for hunters (-0.1) and for forest rangers (0); and good for the system coordinator (1) (Fig 4). This was mostly due to a lack of knowledge of the current objective. Only one hunter stated that the objective was to preserve the officially free status. Four hunters thought the objective was both to protect livestock and to preserve public health; and two hunters did not know about the objective. Forest rangers did not know clearly about the objective as well, thinking that the surveillance was mainly in place to protect livestock.

The acceptability of the operation of the surveillance system was medium for all stakeholders: hunters (0.2), forest rangers (0.17) and for the system coordinator (0.3) (Fig 4).

Hunters were satisfied about their role in the system, which is to report any suspected case of bTB in wildlife (i.e. call forest rangers) or to bring dead-found animals either to forest rangers or to the Faculty of Veterinary Medicine at Liège. They were not satisfied with the consequences of the information flow because a suspicion of bTB in wildlife would potentially create panic in the hunting sector and conflicts with local farmers. One hunter stated that *‘it will led to panic*, *and we have some phobia with this’* (individual interview with hunter, 23^rd^ October 2014). Hunters were afraid of a potential increase of safety measures and controls as well. Nonetheless, they stated that a suspected case could also increase the communication and information sharing. Three out of the seven hunters stated that, if they have the information related to a suspicion, they will increase their vigilance while hunting. Hunters were satisfied with the relations they have with other stakeholders involved in the surveillance, even if they highlighted some issues for the relations with the forest rangers due to administrative constraints.

Forest rangers were satisfied with their intermediate role between hunters and the system coordinator in the surveillance, even if they stated that it was not always easy to collect and to stock dead-found animals. They were unsatisfied with the consequences of the information flow due to the fact that it could increase their workload and that they could be under pressure from hunters especially due to the potential increase of conflicts with farmers. Nonetheless, they stated that a suspicion could help to increase the communication with hunters. Forest rangers were satisfied with the relations they have with stakeholders involved in the bTB surveillance, especially regarding the relations with the system coordinator. Nonetheless, they stated that with hunters it can be sometimes complicated, depending on the hunters: *‘They sometimes get upset quickly*, *whereas we always try to really find compromises to solve some problems’* (individual interview with a forest ranger, 5^th^ November 2014). They also found regrettable the lack of contacts with hunting councils.

The system coordinator was satisfied with her role in the surveillance. She was not completely satisfied with the consequences of the information flow, due to the fact that it could increase conflicts with hunters and increase her workload. Nonetheless, she stated that a suspicion could be useful to collect other relevant data in the field (i.e. information related to the suspicion), and to increase the information sharing from stakeholders. She was also not completely satisfied with the relations she had with other stakeholders involved in the bTB surveillance. She would like to increase the relations with hunting councils. She stated that the relations with hunters were sometimes complex, whereas it was working well with forest rangers. The relations with the FASFC were good even if she found it regrettable that they are not providing her a full hunters’ contact list to be able to contact them when needed.

The trust in the surveillance system was good for hunters (0.5), weak for forest rangers (-0.5) and medium for the system coordinator (0) (Fig 4). For all participants, the critical points in the system are hunters *because ‘hunters do not feel concerned by all this’* (individual interview with a hunter, 23^rd^ October 2014). Limits were highlighted by forest rangers regarding the constraints linked to the transport and storage of dead-found animals. ‘*I think an outbreak will be reported at some time point*. *The problem is an outbreak will sometimes be reported a long time after the start of the initial infection’* (individual interview with the system coordinator, 15^th^ December 2014).

#### Additional information

The use of participatory approaches allowed collecting information related to the context in which surveillance is implemented. Respondents highlighted supplementary issues and proposed also some solutions.

Private veterinarians highlighted problems related to the implementation of SIT also due to the fact that some farmers do not properly restrain their animals. According to them, ways to facilitate the implementation of SIT and the communication with farmers would be to visit farms guided by official inspectors of the FASFC and to have more flexible control measures, without detailing which control measures they were referring to. The increase of financial rewards received by the veterinarians to realise SIT would also beneficial the bTB surveillance in Belgium, as stated by both private veterinarians and by the competent authority responsible for the Sanitary Fund (FPS representative). Private veterinarians working in slaughterhouses also found regrettable the fact that they do not have feedback following their detection of suspicious bTB lesions, which would help them to improve their confidence in the confirmation of suspicious cases.

The national reference laboratory pointed out the lack of historical data regarding previous outbreaks and regarding the strains identified during these outbreaks. The solution for these stakeholders would be to have a data warehouse to store information of suspected cases or outbreaks in a standardised way. They also highlighted the fact that they did not have the origins of the samples to analyse (i.e. mandatory SIT or suspicion in slaughterhouse).

Representatives from the competent authority are expecting a lot of scientific research activities to implement ‘fit-for-purpose’ gamma-interferon tests in the field.

Hunters highlighted problems related to the game processing plants. They stated that when game animal carcases are declared unfit for human consumption they do not have feedback about the reason. One hunter also pointed out that the implementation of some simulation exercises about the detection of a notifiable disease would *‘help everyone to improve their reflexes [to cope with a suspicious case]’* (individual interview with hunter, 4^th^ November 2014). The same hunter proposed to implement field trainings for hunters on infectious diseases.

The forest rangers highlighted the fact that there is a lack of material and resources to be able to transport and to stock dead-found animals to the Faculty of Veterinary Medicine: *‘We do not have gloves or bags resistant enough to safely transport these animals’* (focus group discussion with forest rangers, 5^th^ November 2014).

The system coordinator pointed out the lack of communication with the public health sector. She also stated that an additional information sheet should be provided per suspected case, completed with the requests of supplementary post-mortem analysis by the veterinarian of the field, and sent to the Faculty of Veterinary Medicine with the dead wild animal.

### OASIS flash evaluation

#### Stakeholders involved

A total of 15 stakeholders joined the OASIS flash scoring process: 3 members of the evaluation team and 12 members of the scoring team (Table 4). This full day meeting joined representatives of *(i)* the federal competent authorities (i.e. FASFC, FPS), *(ii)* the national reference laboratory, *(iii)* the veterinary officers at slaughterhouses, *(iv)* the wildlife surveillance coordinator, *(v)* the farmers (president of the European federation of animal health and sanitary safety (FESASS)) and *(vi)* the Scientific Institute of Public Health (WIV-ISP). Among these stakeholders, 6 were also involved in the participatory process: two representatives of the FASFC, one representative of the FPS, two representatives of the national reference laboratory and the wildlife surveillance coordinator.

**Table 4:** Demographics of the stakeholders involved by a full day meeting to score the criteria in the OASIS tool to evaluate the bovine tuberculosis surveillance system of Belgium.

	Stakeholders / Organisations	Number
**Evaluation team**	ANSES	1
	FVM	1
	CIRAD	1
**Scoring team**	FASFC	3
	FPS	1
	National reference laboratory	4
	FESASS	1
	Wildlife surveillance coordinator	1
	Public Health Institute	1
	Veterinary officer of slaughterhouse	1
**Total**		15

ANSES: French agency for food, environmental and occupational health safety; FVM: Faculty of Veterinary Medicine, University of Liège; CIRAD: Centre for agricultural research for developing countries; CVO: Chief Veterinary Officer; FASFC: Federal Agency for the Safety of the Food Chain; FPS: Federal Public Service health, food safety and environment; FESASS: European federation of animal health and sanitary safety.

#### Acceptability assessment

The 78 criteria included in the evaluation tool were scored using the information collected with the questionnaire and on basis of participants’ expert-opinion and experience related to the bTB surveillance.

Based on the scoring of the 19 criteria used to assess the acceptability, results showed that the acceptability of the bTB surveillance system was medium with a score of 62% (criteria scores are compiled using various weights for each criterion) (Table 5).

**Table 5:** Results from the OASIS flash scoring meeting regarding the criteria used for the assessment of the acceptability of the bovine tuberculosis surveillance system of Belgium.

Criteria	Score (/3)
Taking partners’ expectations related to the objective into account	2
Existence of an operational management structure (central unit)	2
Existence of an operational steering structure that is representative of the partners (steering committee)	2
Organization and operations of the system laid down in regulations, a charter, or a convention established between the partners	1
Frequency of meetings of the central coordinating body	3
Active role of intermediate units in the functioning of the system (validation, management, feedback)	3
Adequacy of material and financial resources of intermediary units	3
Existence of coordination meetings at the intermediate level	3
Adequacy of material and financial resources at the field level	0
Effective integration of laboratories in the surveillance system	3
Simplicity of the case or threat definition	2
Simplicity of the notification procedure	3
Simplicity of the data collection procedure	1
Acceptability of the consequences of a suspicion or case for the source or collector of data	0
Adequacy of the data management system for the needs of the system (relational database, etc.)	0
Initial training implemented for all field agents when joining the system	2
Regular reports and scientific publications on the results of the surveillance	2
Feedback of the individual analyses results to field actors	3
Systematic feedback of the assessment results to field actors (excluding news bulletin)	3

Expectations of the majority of the partners regarding the objective of the surveillance system are taken into consideration (score = 2). Nonetheless, it has been highlighted that to be able to protect their farms, farmers are waiting for a better consideration of biosecurity measures in the objectives.

Both components of the surveillance system have an operational management structure (score = 2). There were needs highlighted regarding the clarification of their mandates, but also regarding the coordination between the Regions for wildlife surveillance. There is an existing steering committee (score = 2) with some gaps for a centralised national coordination. Only the positioning of a limited number of partners is framed by an official document (score = 1). Meetings of the central coordinating body (FASFC) are regularly implemented, with a frequency that responses to the needs (score = 3).

The intermediate controlling units (i.e. PCU) have an active role in the implementation of the surveillance (score = 3), and have the adequate material and financial resources (score = 3). Nonetheless, for wildlife surveillance these resources could be improved by the Regions. Coordination meetings at PCU level are regularly organised (score = 3), with focus on bTB. There are shortages of material and financial resources at the funding level (score = 0), especially regarding the weak financial compensation of surveillance testing by the private veterinarians.

The national reference laboratory is effectively integrated in the surveillance system (score = 3).

The case definition is simple, even if there are difficulties related to the interpretation of the skin tests and to the identification of suspicious lesions in slaughterhouses (score = 2). Needs were highlighted regarding the clarification of this case definition for the private veterinarians to be able to know when to report a suspicion. The notification procedure appeared to be simple (score = 3), whereas the data collection procedure appeared to be more complicated to implement (score = 1). Indeed, the SIT is not easy to implement if animals are not well immobilised. The implementation of SIT may vary from farm to farm according to the restraining possibilities in place. The acceptability of the consequences of a suspicion for the source or collector of data is low (score = 0) due to the strict control measures to be implemented in a free status suspended farm (i.e. movement restriction, milk delivery restriction) and to constraints linked to the implementation of follow-up SIT for many years. This acceptability has been defined as very low for farmers, and low for private veterinarians who are in conflicts of interest. Problems were highlighted for wildlife surveillance as well, because some hunters would prefer to bury suspected dead-found animals instead of notifying them.

Currently a single data management system is not in place and epidemiological surveillance data are stored in different databases (score = 0). Nonetheless a request has been made within the FASFC to develop a complete centralised data warehouse where all information about suspicions or outbreaks of all mandatory notified animal diseases is stored.

Only some stakeholders have been trained in the frame of bTB surveillance (score = 2). Private veterinarians have to regularly follow courses, and some hunters have been trained to the basics for suspicion as well. Room for improvement were in the contents and in the frequency of these trainings, especially targeting the private veterinarians.

Regular reports and scientific papers are published, but their number could be increased (score = 2). Improvement could be implemented regarding the frequency of publication and the contents. Regarding the individual analysis, each result is individually communicated to the field actors (score = 3). Regular meetings are also organised at the provincial level in order to share the data obtained from surveillance (score = 3).

### Comparison between the two evaluation processes

The level of acceptability assessed using the participatory methods and tools was 0.23 (on a scale from -1 to +1), which corresponds to 61.5%. The level provided by the OASIS flash assessment was 62%. Both methods provide a similar medium acceptability of the bTB surveillance system of Belgium.

Based on the results of the participatory approaches, three main factors influencing the level of acceptability were detected *(i)* the difficulties for the private veterinarians to fulfil their role regarding SIT and the notification, *(ii)* the lack of hunters’ awareness about the surveillance system, and *(iii)* the lack of resources for forest rangers to be able to collect, to stock and to transport dead-found animals.

Based on the results of the OASIS flash tool, three main factors influencing this level of acceptability were detected *(i)* the weak acceptability of the consequences of notification of a suspicion or confirmed case(s) for farmers (i.e. restrictions on animal movements), *(ii)* the weak financial compensation received of the Sanitary Fund by the private veterinarians to implement prophylactic measures (i.e. SIT), and *(iii)* the difficulties for private veterinarians to implement SIT in farms.

## Discussion

This study allowed us to compare two methods, OASIS flash tool and participatory assessment, to evaluate the acceptability of surveillance systems. Using these two approaches we were able to evaluate the acceptability of the bTB surveillance system of Belgium and to identify several areas for improvement. The level of acceptability was very similar between the two approaches and was considered moderate with a score of 61.5% for the participatory assessment and 62% with OASIS flash approach. As OASIS has been successfully applied for the evaluation of several French surveillance systems [18, 27], this is an indication that the participatory process is also a valuable way to assess the acceptability of surveillance systems.

The comparison between the two approaches was done on the general level of acceptability and on the recommendations provided. However, the comparison in our study was not straight forward. Indeed, most of the indicators used in the OASIS tool (12/19) are also considered in the participatory approaches, but most of the time at a different level. Some other indicators are not considered in the participatory process, and some participatory indicators are not considered in the OASIS tool. Moreover, the scoring process differs from one approach to another. OASIS flash is based on a semi-quantitative scale from 0 to 3; whereas the scoring system for the participatory approaches is based on a semi-quantitative scale of -1 to +1. This highlights the difficulties for comparing the general levels of acceptability obtained from the two evaluations. Thus, careful attention has to be given not to over-interpret the results from this comparison. Nonetheless, by calculating percentages, we were able to provide estimation about how close the results seem to be.

OASIS flash tool is an easy to use tool, providing a questionnaire, a scoring guide and worksheets from which outputs are automatically calculated. Nonetheless, prior knowledge and experience related to surveillance is required from the evaluator [18, 26]. This tool provides an overview of the performances of the surveillance, but does not allow the possibility to modify the evaluation criteria along the evaluation process. The same method is used to assess any type of surveillance, independently of the epidemiological or socio-economical context. For the assessment of the acceptability, when using the Flash version of the evaluation process, there is little involvement of local stakeholders in the process (e.g. farmers, private veterinarians, hunters, forest rangers). Most of the time, there is a restricted number of representatives from local stakeholders in the expert panel. Also, due to the time required for the scoring process, the flash method does not offer the possibility to have open discussions. Indeed, the panel of experts is available for only one day, meaning that the time devoted to the scoring process is limited and that some points may be missing during the discussions. When the complete process of Oasis is followed, a representative panel of local stakeholders are interviewed by the evaluation team in order which helps to have a detailed documentation of the evaluation criteria used.

Even compared to the complete process of an OASIS evaluation, the use of participatory approaches to assess acceptability of the surveillance has the advantage to involve of a higher number of stakeholders in the evaluation, and a higher diversity of the profiles (i.e. farmers, hunters, private veterinarians, etc.). This provides a better view of the surveillance system and leads to context-dependent recommendations. The use of visualisation tools was useful in such a systemic approach as it helped respondents to explain complex ideas and the facilitator to gain and hold the attention of the participants. These tools allowed respondents to discuss about their perception of the current surveillance system and therefore to provide more information about the general context in which surveillance is implemented. Taking into consideration stakeholders’ perceptions and expectations by the participatory approaches in the evaluation framework allowed to develop a relationship of trust with the respondents and to have a better acceptability of the evaluation process itself. Also these approaches are known to be flexible. This advantage allowed the evaluator to adapt the process to the respondents. Nonetheless this process requires time in the field to contact key stakeholders and to organise and implement the interviews, but also time to analyse all obtained information. It requires specific skills related to the use of participation and regarding group facilitation. There may have bias in the respondents’ selection process due to the fact that only stakeholders who are willing to be part of the study can be interviewed, meaning that most of the respondents involved in such study already have some interest regarding animal health issues.

Interpreting the level of acceptability of the bTB surveillance system is strictly influenced by the lack of gold standards to guide the interpretation of the results [26]. Moreover, in most evaluations of surveillance systems, the acceptability is assessed in a qualitative way meaning that no quantitative score or percentage is provided.

Nonetheless, following these two evaluation methods recommendations can be provided to improve the acceptability of the current system. Both processes highlighted important constraints following a bTB outbreak in a farm, meaning that appropriate financial compensations are required. Low financial compensation for private veterinarians and difficulties to implement SIT in farms were also highlighted and restraining systems in farms are required to facilitate their work.

Based on the participatory assessment, other key points were highlighted leading to complementary context-dependent recommendations. The main limitations of the bTB surveillance are the weak trust in the SIT by most stakeholders and the lack of awareness / interest in surveillance of some hunters. The main recommendations to improve this acceptability level would target the private veterinarians for the cattle surveillance, and the forest rangers for the wildlife surveillance. At the front line of the system, they are key actors and some important issues should be addressed in order to help them in fulfilling their role in the surveillance. The acceptability of the private veterinarians could be improved through an involvement of PCU when performing the SIT, which would facilitate the communication with farmers and decrease the pressure exerted on them. It would also be desirable to involve private veterinarians more closely in the follow-up of the surveillance after a suspicion in order to improve their feeling of belonging to the system. Regarding forest rangers, the improvement of the acceptability should be reached through an increase of their material and financial resources to be able to collect, stock and transport dead-found animals. A better communication with hunters and more specifically with hunting councils should also increase the acceptability.

These two evaluation processes can thus be considered as complementary, both having advantages and limitations. They should be implemented according to the surveillance context (i.e. epidemiological, social, economic factors); but also to the evaluation context (i.e. time and resources available, evaluator(s)’ skills). The use of participatory approaches to assess the acceptability provides some added value compared to more ‘classical’ methods such as the OASIS flash tool. Nonetheless, this added value has to be balanced with the evaluation context. Participatory approaches could be used to assess other evaluation attributes, but could also be helpful for the data collection necessary for other tools (e.g. capture-recapture methods). Moreover, due to the fact these approaches provide information related to the context in which surveillance is implemented, they could allow to better understand some outputs of the evaluation process and to result into better recommendations.
